# Short‐term outcomes of typical versus atypical lung segmentectomy by minimally invasive surgeries

**DOI:** 10.1111/1759-7714.13152

**Published:** 2019-08-02

**Authors:** Boheng Xie, Xiao Sun, Yi Qin, Ao Liu, Shuncheng Miao, Wenjie Jiao

**Affiliations:** ^1^ Division of Thoracic Surgery Affiliated Hospital of Qingdao University Qingdao China; ^2^ No.1 People's Hospital Ningyang county China

**Keywords:** Lung segmentectomy, robotic surgery, short‐term outcomes, video‐assisted thoracic surgery

## Abstract

**Background:**

Segmentectomy is increasingly used to resect lung nodules. Robotic‐assisted thoracic surgery (RATS) is considered a safe and practical method for segmentectomy. Few studies have compared robotic surgery and video‐assisted thoracic surgery (VATS) for lung segmentectomy.

**Method:**

We retrospectively examined 215 consecutive patients who underwent typical (88 patients) or atypical (128 patients) segmentectomy by either robotic surgery or VATS. The postoperative characteristics including operation time, blood loss, pneumonia, tumor size, lymph nodes harvested, chest tube duration, prolonged air leak, atrial fibrillation, and postoperative hospital stay were recorded.

**Results:**

A total of 88 patients underwent typical segmentectomy, while 127 patients underwent atypical segmentectomy. A greater number of lymph nodes were resected via RATS than by VATS (13.24 ± 4.84 vs. 11.71 ± 3.89; *P* = 0.018). The operation time for typical segmentectomy was shorter than that for atypical segmentectomy (115.69 ± 22.32 vs. 131.68 ± 22.52; *P* = 0). No significant differences were found between RATS and VATS in terms of chest drainage duration and postoperative hospital stay. The incidence of postoperative complications including prolonged air leak and atrial fibrillation was not significantly different between typical segmentectomy and atypical segmentectomy.

**Conclusion:**

Atypical segmentectomy is more complicated than typical segmentectomy, which may lead to increases in complications and operation time. Robotic surgery was safe and practical for segmentectomy compared to VATS and more lymph nodes could be dissected by RATS without increasing the risk of postoperative complications.

## Introduction

Lobectomy with systematic mediastinal lymph node dissection is considered a standard procedure for early‐stage non‐small cell lung cancer (NSCLC).^1^Due to the increased use of low‐dose helical computed tomography (CT), diagnosis of early‐stage NSCLC has increased. For these patients, lobectomy will result in greater loss of lung tissue and worse quality of life (QOL). Some trials have shown that segmentectomy has similar oncological outcomes to lobectomy for early‐stage NSCLC and results in better QOL.^2^, ^3^, ^4^, ^5^, ^6^ Both robotic surgery and video‐assisted thoracic surgery (VATS) are minimally invasive procedures for lung segmentectomy and can resect more lymph node stations and shorten the postoperative hospital stay compared to the open approach.^7^

Lung segmentectomy can be classified into two types; typical segmentectomy and atypical segmentectomy. Typical segmentectomy includes upper segmentectomy of the left upper lobe, lingulectomy, superior segmentectomy of both sides, and basilar segmentectomy of both sides. Atypical segmentectomy includes resection of individual segments of the upper lobe, middle lobe, or basilar segments. Atypical segmentectomy may be technically feasible but remains challenging due to its complications.^8^, ^9^

Some articles have reported positive outcomes for robotic surgery for lung segmentectomy. The aim of this study was to compare short‐term outcomes between robotic surgery and VATS for different types of lung segmentectomy.

## Methods

### Study population

This was a retrospective cohort study using a prospective data retrieved from the Hospital Information System (HIS). Data consecutive patients that underwent segmentectomy by either VATS or robotic surgery in Affiliated Hospital of Qingdao University from January 2015 to December 2017 were enrolled. This research was approved by the Ethics Committee of Affiliated Hospital of Qingdao University. All operations are all carried out by both approaches were performed by one single professional surgeon. Patients were divided into two groups according to the resected segments: group I, typical segmentectomy; group II, atypical segmentectomy. The exclusion criteria were as follows: combined segmentectomy of different lobes and segmentectomy plus wedge resection of another segment. The characteristics of patients included in the study are shown in Table 1 and the features and pathology of the segmentectomies are shown in Table 2.

**Table 1:** Demographic data for patients

Variable	Robotic surgery	VATS	*P*‐value
Group I			
Sex			
Male	16	21	
Female	26	25	
Age, year median (range)	55 (33–78)	60 (33–75)	0.768
Group II			
Sex			
Male	23	17	
Female	43	44	
Age, year median (range)	59 (35–77)	60 (28–79)	0.609
Pathology			
Benign	6	15	
Metastasis tumor	4	3	
NSCLC			
IA1	51	46	
IA2	37	26	
IA3	6	13	
IB	—	1	
IIB	2	2	
IIA	2	—	
IIIA	—	1	

VATS, video‐assisted thoracic surgery; NSCLC, non‐small cell lung cancer

**Table 2:** Features of segmentectomies

Status	Robotic surgery	VATS
Group I		
S1 + 2 + 3	20	13
S4 + 5	7	10
S6	15	21
S7 + 8 + 9 + 10	—	2
Group II		
S1	7	7
S1 + 2	18	26
S2	15	7
S3	6	7
S1 + 3	8	6
S6 + 8	1	1
S7	3	2
S7 + 8	4	4
S1a + S2	1	—
S4	1	1
S8	2	—

Each patient underwent a preoperative work‐up with routine laboratory tests, a pulmonary function test, chest CT scans, ECG, brain magnetic resonance imaging, bone imaging, and/or positron emission tomography (PET)/CT. Lung function was assessed in each patient. During all operations, the No. 12 lymph node was sent for a rapid section pathology test; if the nodule was positive, a lobectomy was performed. If there was an uncontrolled bleed during robotic‐assisted thoracic surgery (RATS) (often from an artery), the first aim was to stop the bleeding and then perform an angioplasty. If that didn't work, lobectomy would be performed then. Patients who were converted to lobectomy were excluded from the final analysis. All patients were managed in the thoracic surgery ward. Prolonged air leaks (≥6 days), operation time (skin opening to closing), blood loss, the number of lymph nodes harvested, chest tube duration, and the incidence of pneumonia and atrial fibrillation were recorded and analyzed.

### Surgical technique for robotic surgery and VATS

In both approaches, patients were positioned laterally in a folding decubitus position on the operating table with double lumen endotracheal intubation. Each patient underwent fiberoptic bronchoscopy carried out by an anesthesiologist to ensure that the tube was in the correct position.

For VATS segmentectomy, we chose a biportal approach; an auxiliary incision was made at the fourth or fifth intercostal space at the anterior axillary line, and a camera (Karl Storz SE & Co., Tuttlingen, Germany) was placed at the seventh or eighth intercostal space at the mid‐axillary line at a 30° downward angle.

Robotic surgery was performed using the da Vinci Surgical System. The four‐arm approach and CO_2_ insufflation were used. A chest tube was set by using the camera port. The vessels, bronchus, and intersegmental plane were divided sequentially using an endostapler (Ethicon or Covidien). Bioglue (Porcine Fibrin Sealant Kit. Bioseal Biotechnology Co Ltd., Guangzhou, China) was sprinkled on the stump of lung tissue before the wound was closed. Robotic surgery data were continuously collected after the first robotic surgery case. Patient controlled analgesia (PCA) was typically used and Pethidine administered when patients complained of severe pain.

## Statistical analysis

The Statistical Package for the Social Sciences (SPSS) 22 software (IBM Corp, Armonk, NY) was used for data analyses. Continuous variables are presented as means ± standard deviations, and categorical variables are expressed as percentages. For comparisons between the two groups, Student's *t*‐test or the Wilcoxon rank‐sum test were used to compare continuous variables, depending on the normality of distribution. A Chi‐square test or Fisher's exact test was used to compare categorical variables. All statistical tests were two‐sided, with a significance level of 0.05.

## Results

From January 2015 to November 2017, a total of 215 patients who underwent lung segmentectomy by robotic surgery or VATS were enrolled in the study. The surgical characteristics of patients are presented in Tables 1 and 2. The operation time (skin opening to closing) was recorded. No patients were converted to open surgery.

No deaths occurred within 30 days in either group. The overall complication rate was 12.6% in the robotic surgery group and 14.9% in the VATS group, and complications mainly included prolonged air leak (≥6 days), pneumonia, and atrial fibrillation. No severe arrhythmia or postoperative bleeding occurred. All postoperative characteristics are shown in Table 3. Of all 215 patients, 21 patients (nine in group I and 12 in group II) had benign conditions (tuberculosis, granuloma, chronic inflammation, sclerosing hemangioma), and seven patients (five in group I and two in group II) had metastatic tumors. The remaining patients had NSCLC. The pathology and distribution of NSCLC are shown in Table 1. The postoperative outcomes are presented in Table 3.

**Table 3:** Early outcomes for patients in each group

Variation	GROUP I		*P*	GROUP II		*P*	General		*P*	General		*P*
	Robotic surgery	VATS		Robotic surgery	VATS		GROUP I	GROUP II		Robotic surgery	VATS	
Tumor size (mm)	1.30 ± 0.58 (0.4–2.5)	1.55 ± 0.83 (0.4–3.0)	0.093	1.36 ± 0.81 (0.5–5.0)	1.52 ± 0.84 (0.3–4.5)	0.27	1.44 ± 0.73 (0.4–3.0)	1.44 ± 0.82 (0.3–4.5)	0.996	1.34 ± 0.72 (0.3–5.0)	1.53 ± 0.83 (0.3–4.5)	0.061
Blood lost (mL)	47.14 ± 30.14 (10–150)	49.78 ± 60.35 (10–1200)	0.799	52.88 ± 28.65 (10–100)	49.18 ± 46.88 (10–300)	0.589	48.52 ± 48.10 (10–1200)	49.92 ± 36.14 (10–300)	0.809	56.65 ± 29.23 (10–150)	58.79 ± 122.38 (10–1200)	0.502
OP time (skin‐to‐skin) (minute)	119.29 ± 22.8 (55–180)	112.39 ± 21.5 (65–165)	0.149	130.61 ± 26.01 (90–200)	132.87 ± 18.11 (100–190)	0.573	115.69 ± 22.32 (55–180)	131.68 ± 22.52 (90–200)	0	126.20 ± 25.3 (55–200)	124.07 ± 22.05 (65–190)	0.118
lymph nodes harvested Mean ± SD	13.41 ± 4.27 (7–24)	12.21 ± 4.01 (5–21)	0.211	13.15 ± 5.19 (6–24)	11.35 ± 3.8 (5–26)	0.40	12.79 ± 4.16 (5–24)	12.32 ± 4.67 (5–26)	0.484	13.24 ± 4.84 (6–24)	11.71 ± 3.89 (5–26)	0.018
Chest tube(d) Mean ± SD	2.54 ± 1.33 (1–9)	2.61 ± 1.48 (1–9)	0.84	2.74 ± 1.48 (1–10)	2.75 ± 1.53 (1–8)	0.918	2.58 ± 1.40 (1–9)	2.68 ± 1.46 (1–10)	0.625	2.67 ± 1.42 (1–10)	2.69 ± 1.51 (1–9)	0.865
Prolonged air leak (d)	2.4% 1/42	4.34% 2/46	1.00	6.06% 4/66	8.20% 5/61	1.00	3.41% 3/88	8.67% 11/127	0.164	4.63% 5/108	6.54% 7/107	0.569
Atrial fibrillation	7.14% 3/42	2.22% 1/46	0.344	4.55% 3/66	6.55% 4/61	0.710	4.55% 4/88	5.51% 7/127	1.00	5.55% 6/108	4.67% 5/107	1.00
Postoperative hospital stay (d)	4.83 ± 1.74 (4–11)	4.98 ± 1.77 (3–11)	0.704	5.16 ± 1.96 (3–13)	5.36 ± 1.42 (3–11)	0.49	4.91 ± 1.77 (3–11)	5.25 ± 1.71 (3–13)	0.156	5.03 ± 1.87 (3–13)	5.20 ± 1.25 (3–11)	0.48
Pneumonia	2.38% 1/42	4.35% 2/46	1	3.03% 2/66	3.27% 2/61	1	3.41% 3/88	3.14% 4/127	1.00	2.78% 3/108	3.74% 4/107	0.721

VATS, video‐assisted thoracic surgery; OP, operation; D, day

## Treatment efficacy

A total of 88 continuous patients were included in group I, while 127 patients were included in group II. The operation time was longer in group II than in group I (131.69 ± 22.50 minutes vs. 115.68 ± 2.32 minutes; *P* < 0.00001). The number of lymph nodes harvested was not significantly different between groups (12.79 ± 4.16 vs. 12.32 ± 4.67; *P* = 0.484). The chest tube duration and postoperative hospital stay were longer in group II, but the differences were not significant (2.68 ± 1.46 vs. 2.58 ± 1.40 days; *P* = 0.0.625 and 5.25 ± 1.71 vs. 4.91 ± 1.77 days; *P* = 0.156). No differences were found between group I and group II in terms of prolonged air leak and atrial fibrillation (3.41% vs. 8.67%; *P* = 0.164 and 5.51% vs. 4.88%; *P* = 1.00).

At total of 108 continuous patients were included in the robotic surgery group, and 107 patients were included in the VATS group. More lymph nodes were resected in the robotic surgery group than in the VATS group (13.24 ± 4.84 vs. 11.71 ± 3.89; *P* = 0.018). The robotic surgery group had a nonsignificantly longer in operation time than the VATS group (126.20 ± 25.3 minutes vs. 124.07 ± 2.05 minutes; *P* = 0.118).

## Typical segmentectomy

In group I, 46 patients underwent VATS, and 42 patients underwent robotic surgery. The operation time of the VATS group tended to be shorter than that of the robotic surgery group (112.39 ± 21.5 vs. 119.29 ± 22.8 minutes; *P* = 0.149). The number of lymph nodes harvested was greater in the robotic surgery group than in the VATS group (13.41 ± 4.27 vs. 12.21 ± 4.01; *P* = 0.211). The incidence of atrial fibrillation was greater in the robotic surgery group than in the VATS group (7.14% vs. 2.22%) but without a significant difference (*P* = 0.344).

## Atypical segmentectomy

In group II, 61 patients underwent VATS, and 66 underwent robotic surgery. The number of lymph nodes dissected was greater in the robotic surgery group than in the VATS group (13.15 ± 5.19 vs. 11.35 ± 3.8; *P* = 0.40), similar to group I. The operation time for atypical segmentectomy in the robotic surgery group tended to be shorter than that for VATS segmentectomy (130.61 ± 26.01 vs. 132.87 ± 18.11 minutes; *P* = 0.573). No significant difference in postoperative complications was observed between the robotic surgery and VATS groups.

## Discussion

Previous studies have examined the short‐term outcomes of the two microinvasive procedures in lung segmentectomy and showed that robotic surgery was as safe and practical for lung segmentectomies as VATS.^10^ However, few articles have examined the differences between typical and atypical segmentectomy. In this study, patients in the two groups had similar preoperative characteristics. During the study, no deaths occurred within 30 days. No severe complications such as bronchopleural fistula and cardiovascular failure occurred. The main complications were prolonged air leaks, pneumonia, and atrial fibrillation. Two important differences between the two procedures were observed.

The first significant difference was the dissection of lymph nodes; in general and in each group, robotic surgery resulted in resection of a greater number of lymph nodes than VATS. However, in some studies, the number of lymph stations instead of the number of lymph nodes have been documented. Rinieri *et al*. reported that lymph node dissection was greater in robotic surgery (9; range 5–12) than in VATS (6; range 1–8); however, a very limited number (51) of patients were included.^11^ Han *et al*. advised that in 30 cases of single‐port video‐assisted thoracoscopic pulmonary segmentectomy, the number of dissected lymph nodes was 7.7 ± 5.7 (range 0–20).^12^ Li *et al*. reported that more lymph nodes stations may be dissected via robotic surgery than uniportal video‐assisted thoracic surgery (UVATS), but the number of dissected lymph nodes between the two approaches were not significantly different in that article.^13^ Compared to open surgery, robotic surgery and VATS showed more advantages in dissecting more stations of lymph nodes. Some of the researches contain both lobectomy and segmentectomy. Novellis *et al*. also reported that robotic surgery and VATS can dissect a greater number of lymph node stations than open surgery.^7^ Wilson *et al*. reported that robotic resection was superior to VATS in the rate of nodal upstaging.^14^ Krantz *et al*. reported that patients with clinical stage I NSCLC with more lymph nodes assessed demonstrated more nodal upstaging.^15^ Dissection of more lymph nodes may lead to a better prognosis. In our opinion, the reasons that lead to the differences in lymph node resection are that sampling of lymph nodes instead of systematic dissection may be performed if the tumor is at an early stage or in less invasive types such as adenocarcinoma in situ (AIS) or microinvasive adenocarcinoma (MIA). The clearer three‐dimensional vision and stable and omnidirectional mechanical manipulation of robotic surgery can greatly enhance surgical manipulation, which may make a surgeon more confident and increase their willingness to resect lymph nodes in difficult positions that cannot usually be resected using VATS. Robotic surgery is thought to be less likely to lead to uncontrolled bleeding than VATS for lobectomies.^16^ The 30‐day mortality and conversion rate to open surgery were significantly lower in patients who underwent robotic‐assisted segmentectomy/lobectomy than in those who underwent video‐assisted segmentectomy/lobectomy.^17^ However, the number of resected lymph nodes was not significantly different between group I and group II (12.79 ± 4.16 vs. 12.32 ± 4.67; *P* = 0.484).

Another interesting point is the operation time. In general, the operation time of atypical segmentectomy was significantly longer than that of typical segmentectomy (131.69 ± 22.50 vs. 115.68 ± 2.32; *P* < 0.00001). The operation time varied in different studies. Handa *et al*. reported that complex segmentectomy will extend the operation time when compared to simple segmentectomy (180 vs.143.5 minutes; *P* < 0.0001).^18^ Pardolesi A *et al*. documented that segmentectomy via robotic surgery in 17 patients resulted in an average operation time of 180 minutes.^19^ Dylewski *et al*. evaluated a total endoscopic robotic video‐assisted approach in 35 patients, and the average operation time was 189 minutes.^20^ Demir *et al*. reported that robotic surgery required a longer operation time than VATS (76 ± 23 minutes vs. 65 ± 22 minutes; *P* = 0.018).^21^ In typical segmentectomy, VATS resulted in a shorter operation time than robotic surgery (112.39 ± 21.5 vs. 119.29 ± 22.8; *P* = 0.149). However, for atypical segmentectomy, the operation time was slightly shorter for robotic surgery (130.61 ± 26.01 vs. 132.87 ± 18.11; *P* = 0.573). We assumed that typical segmentectomy was less complicated than atypical segmentectomy in terms of anatomy. Nakazawa *et al*. reported that atypical segmentectomy was more complicated than typical segmentectomy.^8^ Atypical segmentectomies may require much more cutting and dissociation than a typical segmentectomy, which would be more suitable for robotic surgery. The surgeon can also block, hold lung tissue with the robotic arms at the same time, and control the camera, while the assistant operates only the suction and stapler. This procedure can overcome the delay due to communication, thus, robotic surgery makes the surgical process more efficient. Additionally, VATS usually requires three surgeons, whereas two surgeons are sufficient for RATS and the special learning curve and lack of palpation appear to be the main disadvantages of robotic surgery.^22^, ^23^

The incidence of postoperative complications was 12.6% for robotic surgery and 14.9% for VATS. Prolonged air leak (≥6 days), pneumonia, and atrial fibrillation were the main complications. The incidence of postoperative complications after segmentectomy via robotic surgery ranged from 10% to 29% in different studies.^20^, ^21^, ^24^, ^25^ In our study, robotic surgery did not exhibit a significant difference in the incidence of complications compared to VATS, and the incidence rates of postoperative complications for typical and atypical segmentectomy were similar. Although atypical segmentectomy is more difficult to perform, it is safe if surgeons are experienced and proficient. The stable omnidirectional manipulation and the enlarged three‐dimensional view can increase the safety of the operation. However, the main disadvantage of robotic surgery was the lack of palpation, and the visual field may not be sufficient if the lung nodule is small and not close to the pleura.

## Limitations

Robotic surgery is equal to, or has some advantages over, VATS, especially for some difficult operations. The learning curves of the two lung segmentectomy procedures are similar. Although robotic surgery has some advantages over VATS, it has limitations: (i). Robotic surgery is more costly than VATS for lung operations, and this should be considered during operation planning.^7^ That may lead to biases in the non‐randomised and retrospective study. (ii) Only a few thoracic surgeons can perform segmentectomy using these two approaches, and few studies comparing the two approaches are available. (iii) Long‐term follow‐up has not been conducted, and it is unknown whether lymph node resection can lead to survival benefits.

In Summary, atypical segmentectomy is more complicated than typical segmentectomy and therefore requires a longer operation time. Robotic surgery is as safe and practical for segmentectomy as VATS and can resect more lymph nodes than VATS, with no increase in the incidence of atrial fibrillation, pneumonia or prolonged air leak; the benefit in lymph node dissection is remarkable. However, the long‐term outcomes are still unknown, and the prognoses for different types of segmentectomy can vary; therefore, large‐scale randomized clinical trials are still needed.

## Disclosure

Dr Jiao discloses no financial relationship with Intuitive Surgical, Ethicon, Covidien and Bioseal Biotech.
